# High‐level expression of ARID1A predicts a favourable outcome in triple‐negative breast cancer patients receiving paclitaxel‐based chemotherapy

**DOI:** 10.1111/jcmm.13551

**Published:** 2018-02-01

**Authors:** Yuan‐Feng Lin, Ing‐Jy Tseng, Chih‐Jung Kuo, Hui‐Yu Lin, I‐Jen Chiu, Hui‐Wen Chiu

**Affiliations:** ^1^ Graduate Institute of Clinical Medicine College of Medicine Taipei Medical University Taipei Taiwan; ^2^ School of Gerontology Healthy Management College of Nursing Taipei Medical University Taipei Taiwan; ^3^ Department of Veterinary Medicine National Chung Hsing University Taichung Taiwan; ^4^ Division of Breast Surgery and General Surgery Department of Surgery Cardinal Tien Hospital Xindian District, New Taipei City Taiwan; ^5^ Division of Nephrology Department of Internal Medicine Shuang Ho Hospital Taipei Medical University New Taipei City Taiwan

**Keywords:** *ARID1A*, chemotherapy, p38MAPK, paclitaxel, triple‐negative breast cancer

## Abstract

Paclitaxel‐based chemotherapy is a common strategy to treat patients with triple‐negative breast cancer (TNBC). As paclitaxel resistance is still a clinical issue in treating TNBCs, identifying molecular markers for predicting pathologic responses to paclitaxel treatment is thus urgently needed. Here, we report that an *AT‐rich interaction domain 1A* (*ARID1A*) transcript is up‐regulated in paclitaxel‐sensitive TNBC cells but down‐regulated in paclitaxel‐resistant cells upon paclitaxel treatment. Moreover, *ARID1A* expression was negatively correlated with the IC_50_ concentration of paclitaxel in the tested TNBC cell lines. Kaplan‐Meier analyses revealed that *ARID1A* down‐regulation was related to a poorer response to paclitaxel‐based chemotherapy in patients with TNBCs as measured by the recurrence‐free survival probability. The pharmaceutical inhibition with p38MAPK‐specific inhibitor SCIO‐469 revealed that p38MAPK‐related signalling axis regulates ARID1A expression and thereby modulates paclitaxel sensitivity in TNBC cells. These findings suggest that *ARID1A* could be used as a prognostic factor to estimate the pathological complete response for TNBC patients who decide to receive paclitaxel‐based chemotherapy.

## 1 INTRODUCTION

Breast cancer is a major public health problem in females worldwide.^1^ The universality and occurrence rate of breast cancer have risen markedly over the past several decades. Of note, triple‐negative breast cancer (TNBC), which lacks the characteristic receptors for oestrogen, progesterone and Her2/neu, is an aggressive tumour that is associated poor survival and represents an important clinical challenge.^2^ Currently, chemotherapy remains the standard breast cancer treatment. However, breast cancers may display chemoresistance and radioresistance.^3^ Paclitaxel, which is an extract of the tree *Taxus brevifolia*, is a potent chemotherapeutic agent used against breast cancers.^4^ Paclitaxel mechanistically stabilizes tubulin polymerization resulting in arrest of mitosis and subsequent apoptosis.^5^ However, paclitaxel has had limited success in cancer therapy because of the activation of cytoprotective signalling pathways, including the nuclear factor kappa B (NF‐κB), phosphatidylinositol 3‐kinase (PI3K)/Akt and mitogen‐activated protein kinase (MAPK) signalling pathways, which induce drug resistance.^6^, ^7^, ^8^ In addition, paclitaxel promotes cell survival and inhibits apoptosis through up‐regulation of antiapoptotic (X‐linked inhibitor of apoptosis protein [XIAP], inhibitor of apoptosis‐1 [IAP‐1], IAP‐2, Bcl‐2 and Bcl‐xL) and proliferative (cyclooxygenase 2, c‐Myc and cyclin D1) proteins.^9^

Chromatin remodelling, which regulates the synthesis, transcription and repair of DNA, is important in cell nuclear activities. Genetic mutation of the chromatin remodelling complex has been identified as a mechanism of tumour occurrence and development.^10^ Here, we analyse the transcriptional profiling of paclitaxel‐sensitive DU4475 and paclitaxel‐resistant MDA‐MB436 without or with paclitaxel treatment, which was determined previously.^11^ We found that the AT‐rich interaction domain 1A (ARID1A) is up‐regulated in DU4475 cells but is down‐regulated in MDA‐MB436 cells. ARID1A is a non‐catalytic subunit of the chromatin remodelling complex and has the ability to combine with DNA or proteins.^12^ Previous studies have demonstrated that ARID1A is a tumour suppressor that is frequently mutated in various cancers, including breast cancer.^13^, ^14^, ^15^ ARID1A loss correlates with mismatch repair deficiency and intact p53 expression in endometrial cancer.^16^ Recently, genetic mutations of ARID1A have been shown to be associated with treatment and prognosis of the tumour.^12^ Mamo et al indicated that low ARID1A RNA or protein expression is related with more aggressive breast cancers.^15^ However, whether such a high mutation rate is associated with the resistance of breast cancer to chemotherapy remains unclear and requires further investigation. Therefore, the aims of this study were to evaluate the effect of the ARID1A gene on breast cancer following paclitaxel treatment and to investigate the possible mechanism. The results suggest that ARID1A may be used to predict the outcome in breast cancer patients receiving paclitaxel‐based chemotherapy. ARID1A thus warrants further investigation as a potential diagnostic and therapeutic marker for breast cancer.

## 2 MATERIALS AND METHODS

### 2.1 Cell lines and cell culture condition

Breast cancer cell lines MDA‐MB‐231 were cultured in Leibovitz's (L‐15) medium (Gibco Life Technologies, Grand Island, NY, USA) supplemented with 10% foetal bovine serum (FBS, Invitrogen) and incubated at 37°C with free gas exchange with atmospheric air. Breast cancer cell lines HCC1143, HCC1806, HCC1937, HCC38 and HCC70 were cultured in RPMI‐1640 medium (Gibco Life Technologies) with 10% FBS and incubated at 37°C with 5% CO_2_. BT‐20 cells were cultured in Eagle's Minimum Essential Medium with 10% FBS and incubated at 37°C with 5% CO_2_. All cell lines were obtained from American Type Culture Collection. All cells were routinely authenticated on the basis of short tandem repeat analysis, morphologic and growth characteristics and mycoplasma detection.

### 2.2 Reverse transcriptase‐polymerase chain reaction

Total RNA was extracted from cells using TRIzol extraction kit (Invitrogen). Aliquots (5 μg) of total RNA were treated with M‐MLV reverse transcriptase (Invitrogen) and then amplified with Taq‐polymerase (Protech) using paired primers (for *ARID1A*, forward‐GCCAGACTCCATATTACAACCAGC and reverse‐GGAATAGGCAGTTTGCTGGGACTG; for glyceraldehyde‐3‐phosphate dehydrogenase (GAPDH), forward‐AGGTCGGAGTCAACGGATTTG and reverse‐GTGATGGCATGGACTGTGGTC).

### 2.3 Western blot analysis

The protein concentrations of total cell lysates, nuclear and cytoplasmic extracts were determined by the Bradford assay (Bio‐Rad, Hercules, CA, USA) using bovine serum albumin as a standard. Samples containing equal quantity of proteins were mixed in Laemmli sample buffer (62.5 mmol/L Tris [pH 6.7], 1.25% SDS, 12.5% glycerol, and 2.5% β‐mercaptoethanol) and boiled for 10 minutes at 100°C before being separated by electrophoresis on 8%‐15% SDS‐PAGE gels and transferred to polyvinylidene difluoride membrane (Millipore, Temecula, CA, USA). After blocking with 5% dry milk in PBST, membranes were explored with antibodies against p‐p38/p38/ARID1A (Cell Signaling, Danvers, MA, USA) and GAPDH (AbFrontier, Seoul, Korea). The horseradish peroxidase‐conjugated secondary antibody incubation was performed, and the specific immunoreactive protein complexes were detected by using the enhanced chemiluminescense method (Amersham Bioscience, Tokyo, Japan).

### 2.4 MTT assay

Cells (1 × 10^5^/mL) were seeded into a 96‐well culture plate. After the incubation with paclitaxel (Aldrich‐Sigma) in the absence or presence of pharmaceutical inhibitors including SCIO‐469 (Tocris Bioscience, Bristol, UK), 10 μL of MTT (3‐(4,5‐dimethylthiazol‐2‐yl)‐2,5‐diphenyltetrazolium bromide) (Molecular Probe, Invitrogen, CA, USA) stock solution was added into each well. The conversion of MTT to formazan by viable cells was performed at 37°C for another 4 hours. After the reaction, 100 μL of DMSO solution was added into each well to solubilize the formazan precipitates. The levels of formazan were determined by optical density at 540 nm using an ELISA reader for calculating cell survival rates.

### 2.5 Microarray and RNA sequencing data processing

Microarray results with accession numbers GSE50832, GSE22513 and GSE32646 and the related clinical data were obtained from the Gene Expression Omnibus database on the NCBI website. Affymetrix DAT files were processed using the Affymetrix Gene Chip Operating System to generate .CEL files. The raw intensities in the .CEL files were normalized by robust multichip analysis, and fold‐change analysis was performed using GeneSpring GX11 (Agilent Technologies). Relative mRNA expression levels were normalized by the median of all samples and presented as log_2_ values. The processed data of microarray and RNA sequencing (RNA‐Seq) for the *ARID1A* gene and the clinicopathological information of breast cancer patients deposited in The Cancer Genome Atlas (TCGA) database were downloaded from the Cancer Browser website. The downloaded microarray and RNA‐Seq results were further normalized by the median of all samples prior to presentation as a boxplot or performing the Kaplan‐Meier (K‐M) analysis.

### 2.6 Kaplan‐Meier analyses

The SurvExpress, K‐M Plotter and TCGA databases contain 1901, 3951 and 437 breast cancer patients, respectively, with follow‐up time intervals. The data were used to estimate the prognostic significance of the *ARID1A* transcript under the condition of recurrence‐free survival (RFS) probability using a K‐M analysis. Moreover, the 195 and 400 breast cancer patients receiving post‐operative chemotherapy from SurvExpress and TCGA databases, respectively, were recruited to perform another K‐M analysis for the *ARID1A* transcript under the condition of RFS probability.

### 2.7 Univariate and multivariate analyses

The 400 breast cancer patients who received post‐operative chemotherapy from the TCGA database were used to perform univariate and multivariate analyses using Cox regression tests. *ARID1A* expression levels and clinical data, including age, pathological stage, T and N, were input as variables for the Cox regression test using RFS conditions.

### 2.8 Establishment of meta‐analysis

A global meta‐analysis of the *ARID1A* transcript was performed using the PrognoScan database which includes public microarray data sets with clinical annotation of gene expression and prognosis from GEO, ArrayExpress and individual laboratory resources. The correlation between *ARID1A* expression and survival in various types of cancers was analysed using the PrognoScan database (http://www.abren.net/PrognoScan/).^17^ The data sets with statistical significance (*P* < .05) in the Cox regression test were downloaded from the PrognoScan website. Different probe identities of *ARID1A* in each data set are presented in Figures 4E and S3. The clinical data and hazard ratio with 95% confidence intervals from different probe identities of *ARID1A* in each data set are shown as tables and forest plots, respectively, and are presented in Figures 4E and S3.

### 2.9 In silico analysis

Genes with a 1.5‐fold change threshold relative to control cells in DU4475 and MDA‐MB436 cells post‐treated with paclitaxel at 10× IC_50_ concentrations were uploaded to the Ingenuity Pathway Analysis (IPA) website (Ingenuity Systems, www.ingenuity.com). Data from computational predictions for the activation or inhibition status of upstream regulators were then output as a text file. Consensus upstream regulators with significant (*P* < .05) *z*‐scores from an in silico analysis of paclitaxel‐treated DU4475 and MDA‐MB436 cells were analysed in a PivotTable report and plotted as a dotplot using Microsoft Excel.

### 2.10 Statistical analyses

SPSS 17.0 software (Informer Technologies, Roseau, Dominica) was used to analyse statistical significance. Paired t‐tests were utilized to compare *ARID1A* gene expression in breast cancer tissues. Pearson's test was performed to estimate the association among *ARID1A*,* VMP1*/*MIR21* mRNA and Paclitaxel IC_50_ (50% of inhibitory concentration) concentrations in the panel of breast cell lines. Survival probabilities were determined by K‐M analysis and log‐rank tests. One‐way ANOVA with Tukey's test was used to estimate the difference in mRNA levels of *ARID1A* and *VMP1*/*MIR21* in DU4475 and MDA‐MB436 cells after paclitaxel treatment. Mann‐Whitney *U*‐tests were used to analyse non‐parametric data. *P* values <.05 in all analyses were considered statistically significant.

## 3 RESULTS

### 3.1 *ARID1A* is predominantly repressed upon paclitaxel treatment in paclitaxel‐resistant breast cancer cells

**Figure 1:**
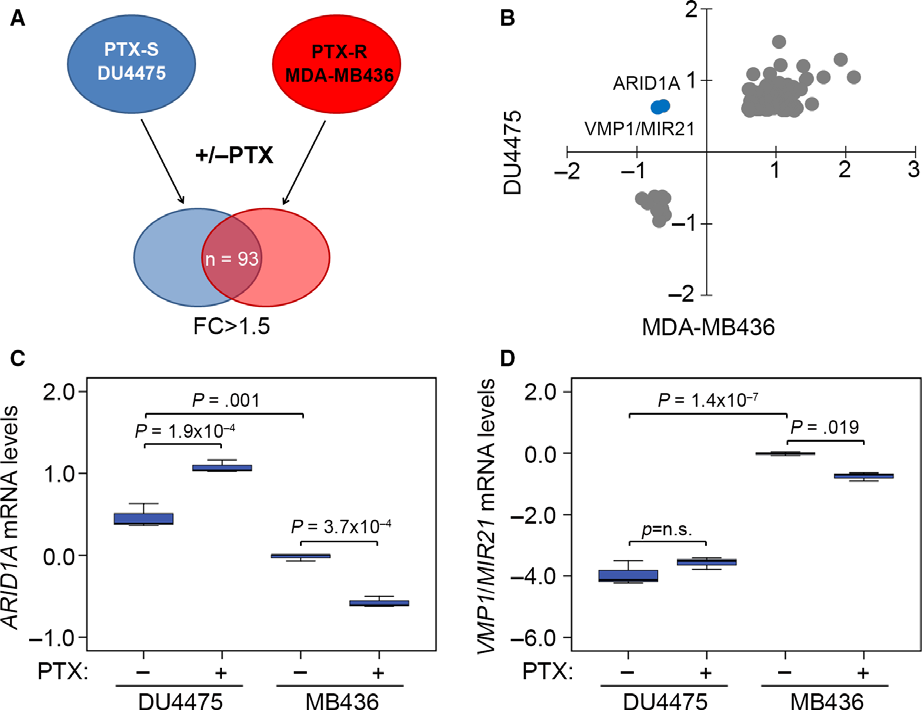
*ARID1A* down‐regulation predicts a poor response to paclitaxel (PTX) treatment in breast cancer cells. (A) A flowchart of identifying consensus genes with 1.5‐fold change (FC) post‐treatment with PTX at the concentration of 10× IC_50_ for 24 h in PTX‐sensitive (PTX‐S) DU4475 cells and PTX‐resistant (PTX‐R) MDA‐MB436 cells. (B) The dotplot for the mRNA levels (log_2_) of 93 consensus genes identified as the strategy shown in A. (C and D) The mRNA levels of *ARID1A* (C) and *VPM1/MIR21*(D) DU4475 and MDA‐MB436 cells post‐treatment without or with PTX at the concentration of 10× IC_50_ for 24 h. Data from three independent experiments were shown in median ± SD. The statistical differences were analysed by one‐way ANOVA using Turkey's test

We first compared the gene expression profiles in paclitaxel‐sensitive DU4475 and paclitaxel‐resistant MDA‐MB436 breast cancer cell lines^11^ without or with paclitaxel treatment at their respective 10‐fold IC_50_ concentrations for 24 hours (Figure 1A). We found 93 consensus genes with 1.5‐fold changes after paclitaxel treatment in DU4475 and MDA‐MB436 cells (Table S1). Our data showed that *ARID1A* (probe ID = 210649_s_at) and *VMP1/MIR21* (probe ID = 224917_at) are predominantly up‐regulated in DU4475 cells but down‐regulated in MDA‐MB436 cells post‐treatment with paclitaxel (Figure 1B‐D). Different from probe 207591_s_at, the other two probes 212152_x_at and 218917_s_at for *ARID1A* detection in the microarray analysis (GSE50832)^11^ yielded similar results (Figure S1A‐C). Furthermore, concerning the detection results obtained using probe 224917_at, the expression of *VMP1/MIR21* detected by probe 220990_s_at was shown to be elevated in DU4475 cells but repressed in MDA‐MB436 cells after paclitaxel treatment (Figure S1D).

**Figure 2:**
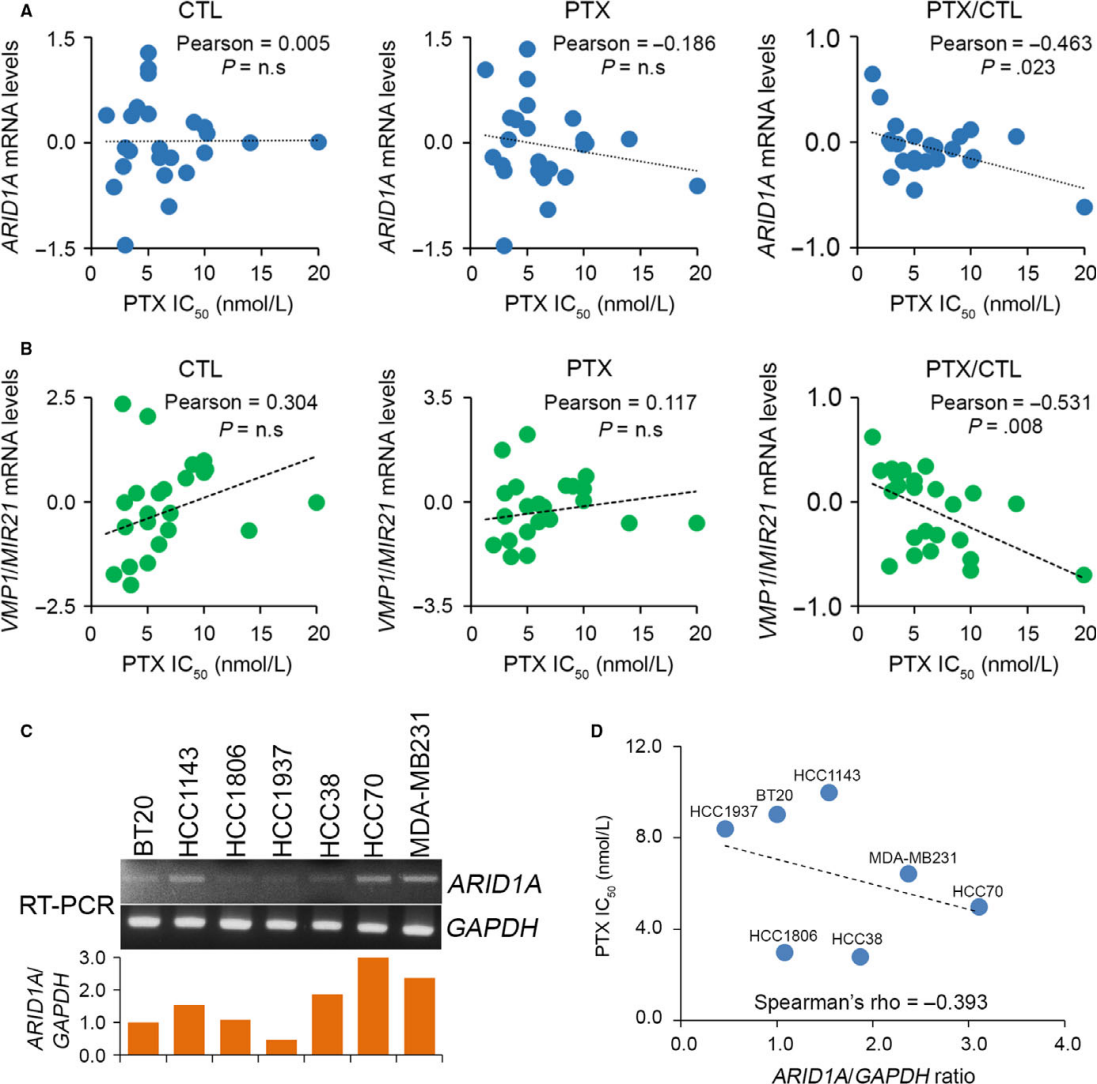
*ARID1A* and *VMP1/MIR21* negatively correlates with paclitaxel (PTX) IC_50_ concentrations post‐treatment with PTX at IC_50_ concentrations for 24 h in a panel of breast cancer cell lines. (A and B) Correlations among *ARID1A* and *VMP1/MIR21* mRNA level and PTX IC_50_ concentration in the tested breast cancer cell lines. The statistical significance of correlations was analysed using Pearson's test. The symbol “n.s” denotes not significant. Each dot in the dotplot indicates the median of mRNA levels from three independent experiments. (C) RT‐PCR analysis for *ARID1A* and *GAPDH* transcripts in TNBC cell lines (*top*). The levels of *ARID1A* transcript were normalized by comparing with the respective *GAPDH* level in the tested cell lines and shown as ratios (*bottom*). (D) Correlates between the normalized *ARID1A* levels and PTX IC_50_ concentrations in a panel of TNBC cell lines. Spearman's correlation test was used to evaluate the statistical significance

Next, we evaluated the correlation between the IC_50_ concentrations of paclitaxel and the mRNA levels of *ARID1A* and *VMP1/MIR21* in a panel of breast cell lines; AU565, BT‐20, BT474, BT‐549, CAL‐51, DU4475, HCC‐1143, HCC‐1419, HCC‐1428, HCC‐1500, HCC‐1806, HCC‐1937, HCC‐1954, HCC‐38, HCC‐70, Hs578T, MCF‐7, MDA‐MB‐231, MDA‐MB‐436, MDA‐MB‐468, SKBR3, T47D and ZRT post‐treatment with or without paclitaxel treatment at their respective 10‐fold IC_50_ concentrations. Whereas the *ARID1A* and *VMP1/MIR21* mRNA levels detected by different probes in the microarray analysis (GSE58032) did not appear to be significantly correlated, the changes in *ARID1A* detected by different probes, except for probe 207591_s_at, in paclitaxel‐treated cells compared to untreated groups were negatively correlated with the respective paclitaxel IC_50_ concentrations (Figures 2A and S2A‐C). Whereas the mRNA levels of *VMP1/MIR21* detected by probe 224917_at were shown to be positively correlated, the *VMP1/MIR21* mRNA levels detected by probe 220990_s_at were negatively correlated with the paclitaxel IC_50_ concentrations of each tested cell line (Figures 2B and S2D). However, the changes of *VMP1/MIR21* mRNA after paclitaxel treatment were inversely correlated with the respective paclitaxel IC_50_ concentrations in the detected breast cancer cells (Figures 2B and S2D). Because *ARID1A* down‐regulation in paclitaxel‐resistant cells and alteration upon paclitaxel treatment is more dominant than *VMP1/MIR21*, we next validated the correlation between endogenous *ARID1A* levels and paclitaxel IC_50_ concentrations in a panel of breast cancer cell lines and thereafter determined the clinical relevance of *ARID1A* in patients with breast cancer. Accordingly, our data showed that the endogenous mRNA levels of *ARID1A* (Figure 2C) are negatively correlated with paclitaxel IC_50_ concentrations in tested breast cancer cells (Figure 2D).

### 3.2 *ARID1A* expression is predominantly down‐regulated in the majority of basal‐like breast cancer tissues

**Figure 3:**
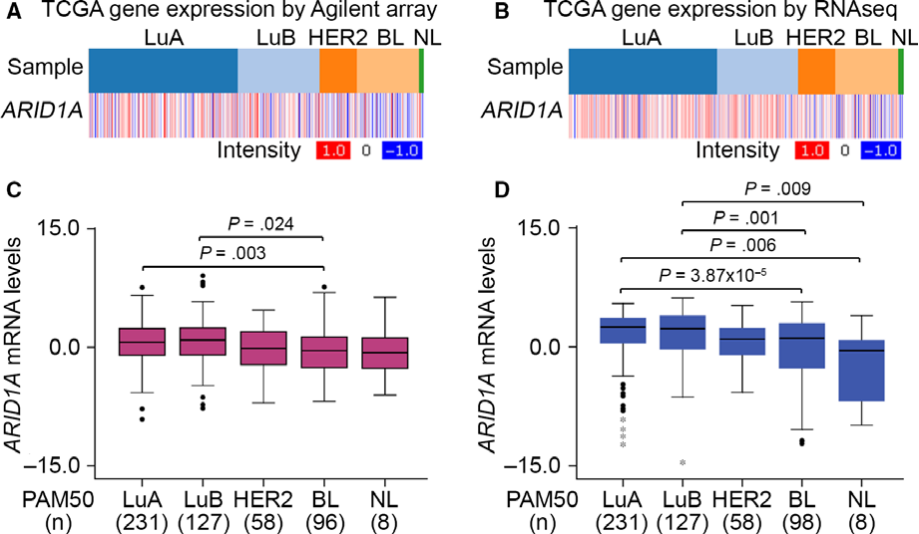
*ARID1A* expression is up‐regulated in basal‐like breast cancer compared to other breast cancer subtypes derived from patient with breast cancer. (A and B) Heatmap for the transcription profiling of *ARID1A* using microarray (A) and RNA‐sequencing (RNAseq) (B) techniques in different cancer types, including luminal A (LuA), luminal B (LuB), HER2, basal‐like (BL) and normal‐like (NL), derived from patients with breast invasive carcinoma using TCGA database. (C and D) Boxplot for the mRNA levels of *ARID1A* analysed by microarray (C) and RNA‐sequencing (RNAseq) (D) methods in various types of breast cancer from TCGA database. The statistical differences were analysed by one‐way ANOVA using Turkey's test

We next dissected the transcriptional profiling of *ARID1A* across all breast cancer subtypes using the TCGA database.^18^ We made use of Agilent microarray and RNA sequencing to analyse *ARID1A* gene expression (Figure 3A‐D). In the Agilent microarray analysis, tumours with a high *ARID1A* signature were enriched in the luminal A and luminal B subgroups compared to the basal‐like subtype (Figure 3A,C). Based on the RNA‐Seq methods, the *ARID1A* mRNA levels in luminal A and luminal B groups were higher than those of basal‐like and normal‐like subtypes (Figure 3B,D).

### 3.3 *ARID1A* down‐regulation predicts a significantly shorter RFS in breast cancer

**Figure 4:**
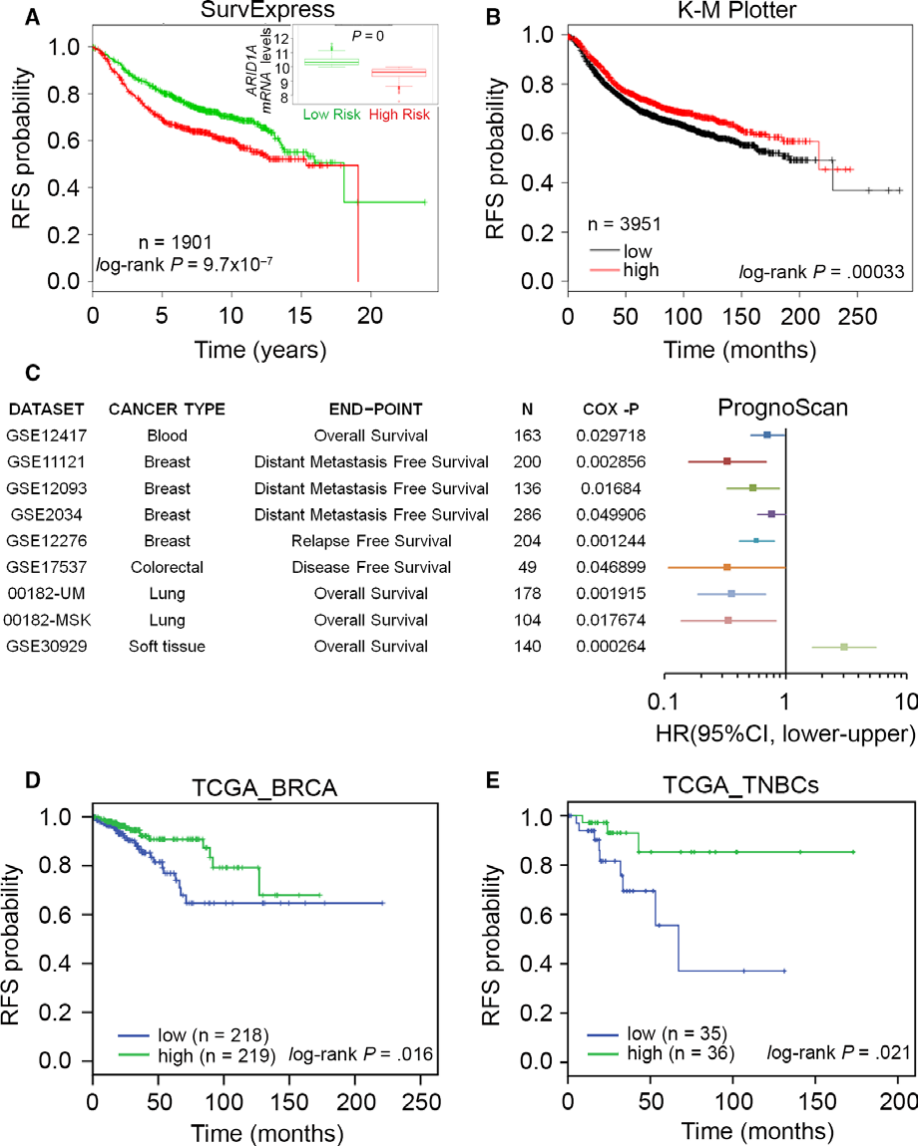
*ARID1A* down‐regulation refers to a poor recurrence‐free survival (RFS) rates in breast cancer patients. (A) Kaplan‐Meier analysis for *ARID1A* expression under the condition of RFS probability in breast cancer patients using SurvExpress database. HR denotes hazard ratio. Insert, boxplot for the mRNA levels of *ARID1A* in high (green) and low (red)‐risk cohorts in A. (B) Kaplan‐Meier analysis for *ARID1A* expression under the condition of RFS probability in patients with breast cancer using K‐M Plotter database. (C) A global meta‐analysis of *ARID1A* expression using PrognoScan database. HR denotes hazard ratio. (D and E) Kaplan‐Meier analysis for *ARID1A* expression under the condition of RFS probability in unclassified breast invasive carcinoma (BRCA) patients (D) and clinical cohort with TNBCs (E) using TCGA database

To further validate the potential prognostic significance of our findings, we researched publicly available platforms of expression analysis, including SurvExpress,^19^ K‐M Plotter^20^ and PrognoScan.^17^ First, we estimated the prognostic significance of *ARID1A* in predicting the RFS rates, which frequently reflect chemotherapeutic responses in breast cancer patients. From the SurvExpress database, the prognostic value of low *ARID1A* mRNA expression was significantly correlated with poor RFS rates in 1901 breast cancer patients (Figure 4A). Furthermore, the mRNA levels of *ARID1A* in the high‐risk cohort were significantly down‐regulated compared to the low‐risk cohort in breast cancer patients (Figure 4A). Accordingly, *ARID1A* down‐regulation appeared to be associated with unfavourable RFS rates in breast cancer patients, based on the K‐M Plotter database (Figure 4B). Similar results were also observed in different *ARID1A* probes within the K‐M Plotter database against breast cancer patients (Figure S3). We found that patients with higher *ARID1A* expression levels were more likely to have a favourable RFS rate. Next, we performed a global meta‐analysis for *ARID1A* using the PrognoScan database. Using a Cox *P*‐value of <.05 (Figure 4C), lower *ARID1A* expression in blood, breast, lung and colorectal cancers, with the exception of soft tissue cancer, was associated with a poorer outcome. Particularly in breast cancer, *ARID1A* down‐regulation was highly correlated with cancer progression, for example, metastasis and recurrence (Figure 4C). Using TCGA database, we found that *ARID1A* down‐regulation refers to a poor RFS probability in unclassified breast invasive carcinoma (BRCA) patients (Figure 4D). Significantly, *ARID1A* down‐regulation was appeared to strongly predict an unfavourable RFS rate in patients with TNBC in TCGA database (Figure 4E).

### 3.4 ARID1A up‐regulation predicts a favourable response to paclitaxel‐based chemotherapy in breast cancer patients

**Figure 5:**
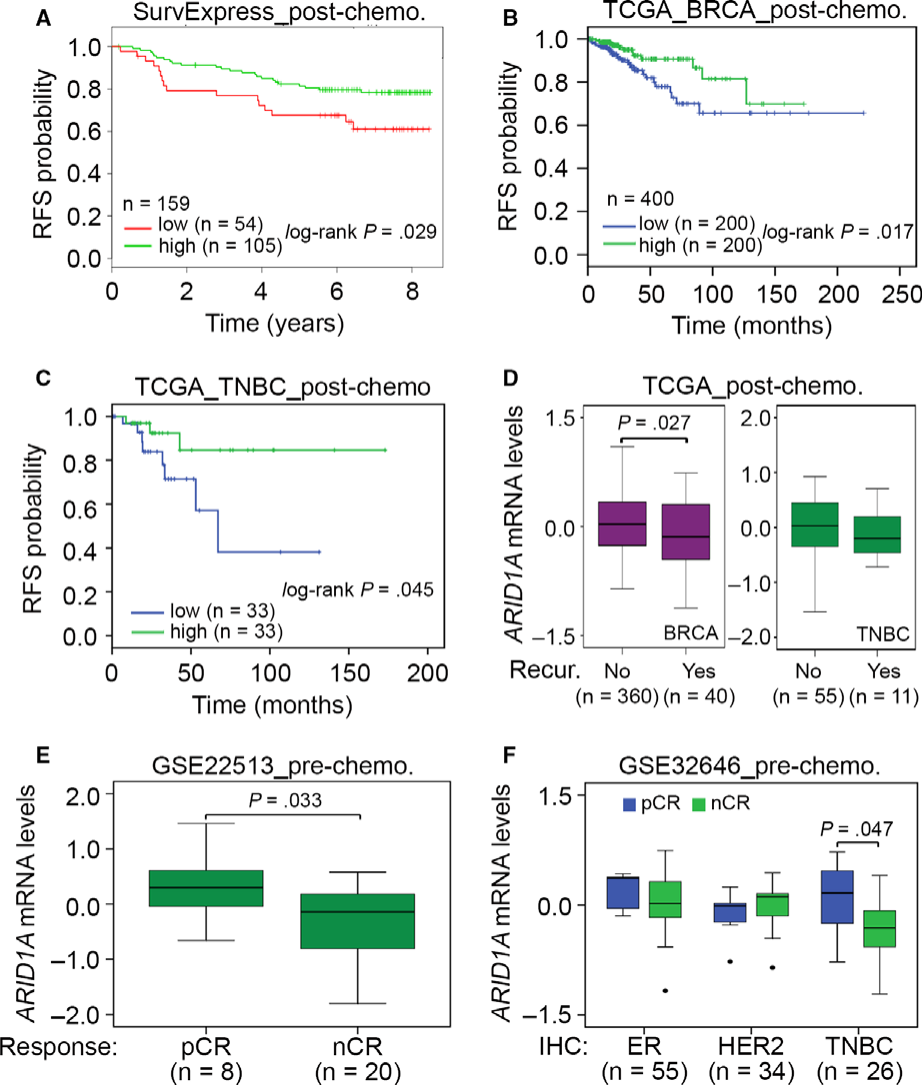
*ARID1A* down‐regulation predicts a poor response to paclitaxel (PTX) chemotherapy in breast cancer patients. (A‐C) Kaplan‐Meier analysis for *ARID1A* expression under the condition of RFS probability in unclassified breast cancer patients and TNBC cohort with post‐operative chemotherapy using SurvExpress (A) and TCGA (B and C) databases. (D) Boxplot for mRNA levels of *ARID1A* in tumour biopsy derived from unclassified breast cancer patients and TNBC cohort receiving post‐operative chemotherapy without (No) or with (Yes) cancer recurrence using TCGA database. The statistical difference was analysed by *t* test. (E) Boxplot for mRNA levels of *ARID1A* in tumour biopsy derived from breast cancer patients with PTX pretreatment using GSE22513 data set. The statistical difference was estimated by non‐parametric Mann‐Whitney test. (F) Boxplot for mRNA levels of *ARID1A* in breast cancer tissues derived from ER(+), HER2(+) and triple‐negative, which identified by immunohistochemistry (IHC) analysis, breast cancer patients who received pre‐operative chemotherapy (pre‐chemo.) using GSE32646 data set. The statistical differences were analysed by one‐way ANOVA using Turkey's test. In (E) and (F) pCR and nCR denote pathological complete response and no pathological complete response, respectively

We further evaluated the prognostic significance of *ARID1A* in breast cancer patients who received paclitaxel‐based chemotherapy. Using SurvExpress and TCGA databases, we found that *ARID1A* up‐regulation reflected a favourable prognosis under the condition of RFS probability in unclassified BRCA patients and TNBC cohort receiving post‐operative paclitaxel‐based chemotherapy (Figure 5A‐C). Cox regression tests also showed that *ARID1A* up‐regulation significantly predicted a good prognosis under the crude and adjusted hazard ratio determination using univariate and multivariate models, respectively, in TNBC patients receiving post‐operative chemotherapy (Table 1). In addition, we analysed *ARID1A* expression in breast cancer patients receiving paclitaxel‐based chemotherapy. The results revealed that the expression in unclassified BRCA and TNBC patients with cancer recurrence was significantly lower than that in patients with no recurrence (Figure 5D). Neoadjuvant chemotherapy (NAC) is used in breast cancer treatment of downstage tumours.^21^ We analysed the transcriptional profile of *ARID1A* in breast tumours derived from breast cancer patients receiving preoperative paclitaxel‐based NAC by using GSE22513 data set.^22^, ^23^, ^24^ *ARID1A* mRNA levels in breast tumours derived from patients with no pathological complete response (nCR) were significantly lower than those from patients with pathological complete response (pCR) (Figure 5E). Moreover, using GSE32646 data set,^25^ among patients receiving paclitaxel‐based NAC, *ARID1A* mRNA levels in TNBC, but not breast tumour of ER(+) and HER2(+), derived from patients with nCR were significantly (*P *<* *.05) decreased compared to those of patients with pCR (Figure 5F).

**Table 1:** Cox univariate and multivariate analyses under the condition of RFS probability in association with ARIDIA mRNA expression levels and pathological stage derived TCGA cohort with 71 TNBCs

RFS				
Variables	Crude HR (95% CI)	*P*	Adjusted HR (95% CI)	*P*
Age				
62<	1	NA	1	NA
62≥	3.49 (1.08‐11.3)	.037	1.50 (0.33‐6.78)	.60
Pathologic stage				
I‐IIA	1	NA	1	NA
IIB‐IV	2.16 (0.68‐6.83)	.189	0.40 (0.04‐3.74)	.423
pT				
T1‐T2	1	NA	1	NA
T3‐T4	2.88 (0.85‐9.72)	.088	10.4 (0.98‐111.6)	.052
pN				
N0‐N1	1	NA	1	NA
N2‐N3	4.41 (1.28‐15.1)	.019	7.30 (0.89‐60.1)	.065
ARID1A expression				
Low	1	NA	1	NA
High	0.24 (0.06‐0.89)	.033	0.11 (0.02‐0.63)	.013

RFS, recurrence‐free survival; TNBC, triple‐negative breast cancer; TCGA, The Cancer Genome Atlas; ARID1A, AT‐rich interaction domain 1A.

### 3.5 p38 mitogen‐activated protein kinase‐related pathways underlying paclitaxel resistance in TNBC cells

**Figure 6:**
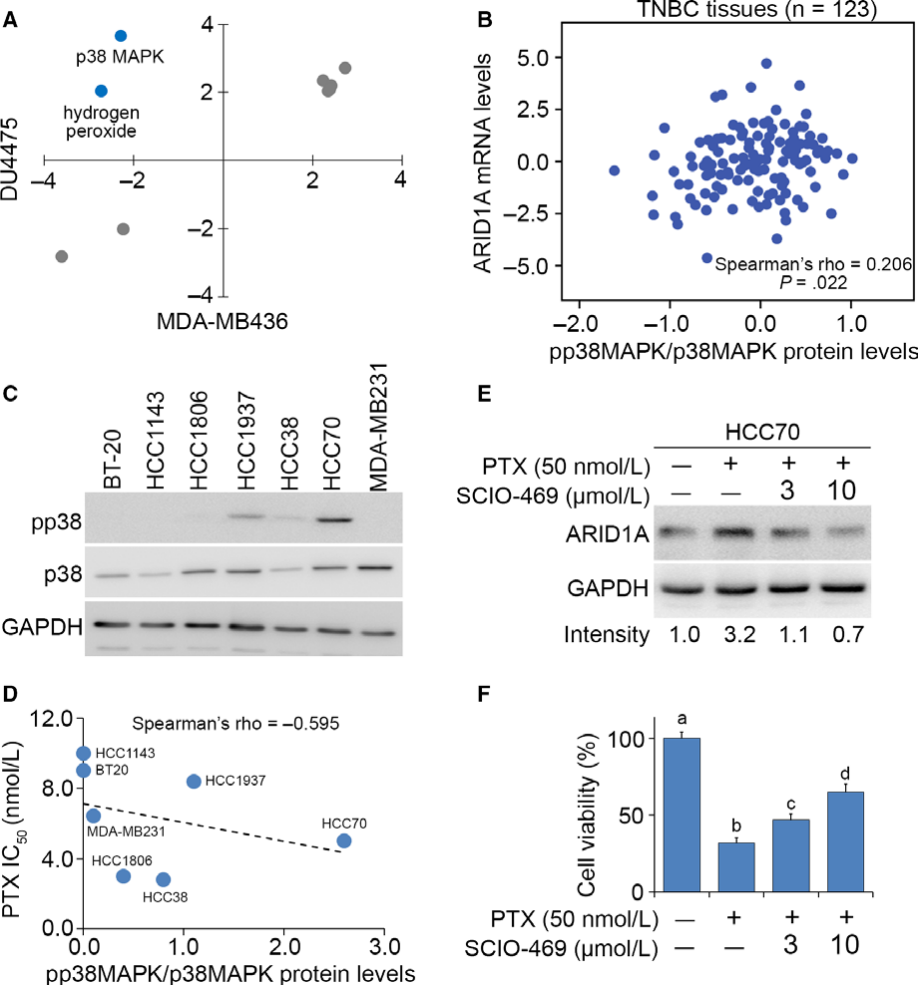
Possible mechanism underlying paclitaxel (PTX) resistance in breast cancer cells. (A) The in silico analysis of consensus upstream regulators that are possibly activated or inhibited after PTX treatment at the concentration of 10× IC_50_ for 24 h in DU4475 and MDA‐MB436 cells using Ingenuity Pathway Analysis software. (B) Correlation between *ARID1A* mRNA levels and the protein levels of phosphorylated p38MAPK (pp38 MAPK) which was normalized with total p38MAPK protein levels using TCGA database. (C) Western blot analysis for pp38MAPK (pp38), p38MAPK (p38) and GAPDH proteins in the tested TNBC cell lines. (D) Correlation between PTX IC50 concentrations and normalized pp38MAPK protein levels in the tested TNBC cells lines. In (B and D) the statistical significance of correlations was analysed by using Spearman's test. (E) Western blot analysis for ARID1A and GAPDH proteins in HCC70 cells pre‐treated without or with p38MAPK inhibitor SCIO‐469 at 3 or 10 μmol/L prior to the treatment without or with PTX at 50 nmol/L for 24 h. In (C and E) GAPDH was used as an internal control of protein loading. (F) Cell viability of HCC70 cells that was treated by the procedure as shown in E. The different letters represent the statistical significance at *P* < .05 in non‐parametric Mann‐Whitney test

We next performed an in silico analysis using IPA software to predict potentially activated/inhibited upstream regulators related to the mechanism of paclitaxel resistance in breast cancer cells. As shown in Figure 6A and Table S2, p38 mitogen‐activated protein kinase (p38MAPK) and hydrogen peroxide were up‐regulated in DU4475 cells but down‐regulated in MDA‐MB436 cells. Using TCGA database, we found that *ARID1A* mRNA levels in TNBCs positively correlate with the protein levels of phosphorylated p38MAPK (pp38MAPK) which is known as an activated protein form of p38MAPK (Figure 6B). Moreover, our data showed that the protein levels of pp38MAPK in a panel of TNBC cell lines (Figure 6C) inversely correlate with paclitaxel IC50 concentrations (Figure 6D). Significantly, the pretreatment with p38MAPK inhibitor SCIO‐469^26^ dose dependently suppressed the enhanced ARID1A protein levels by paclitaxel in HCC70 cells that are relative sensitive to paclitaxel treatment (Figure 6E). In contrast, the pretreatment of SCIO‐469 significantly (*P* < .05) promoted the paclitaxel resistance in HCC70 cells (Figure 6F).

## 4 DISCUSSION

Chemoresistance, the main obstacle in cancer therapy, is caused by the onset of drug‐resistant cells in cancer tissues.^27^ Currently, the standard treatment for TNBCs is cytotoxic chemotherapy. However, some patients with TNBCs are highly chemotherapy resistant and relapse quickly after treatment in the adjuvant setting.^21^ Paclitaxel is the first‐line chemotherapeutic drug for clinical treatment of TNBCs. However, drug resistance often appears chemotherapy.^28^ We have recently described a novel gene controlling paclitaxel resistance in TNBCs. We analysed the transcriptional profiles of paclitaxel‐sensitive DU4475 and paclitaxel‐resistant MDA‐MB436 with or without paclitaxel treatment (Figure 1A,B). In the present study, *ARID1A* and *VMP1/MIR21* were found to be up‐regulated in DU4475 cells but down‐regulated in MDA‐MB436 cells. *ARID1A* mRNA levels were altered more dramatically upon paclitaxel treatment in both DU4475 and MDA‐MB436 cells compared to *VMP1/MIR21* (Figures 1C,D and S1). Recent evidence shows that *ARID1A* gene silencing reduces the sensitivity of ovarian cancer to cisplatin via the regulation of AKT expression. Moreover, down‐regulation of ARID1A decreased the levels of apoptosis in cancer cells and induced resistance to the killing effect of cisplatin.^29^ Our current data indicate that IC_50_ concentrations of paclitaxel are inversely correlated with *ARID1A* and *VMP1/MIR21* mRNA levels (Figures 2 and S2). *ARID1A* down‐regulation predicts a poor response to paclitaxel chemotherapy in TNBC patients (Figure 5). *ARID1A* mRNA levels in TNBC tumours derived from patients with nCR are significantly lower than that of patients with pCR (Figure 5E). However, it was recently demonstrated that ARID1A is recruited to DNA double‐strand breaks via ataxia telangiectasia mutated/ATM‐ and RAD3‐related (ATM/ATR) signalling, where it facilitates the processing of DNA lesions. ARID1A plays a key role in regulating the DNA damage checkpoint.^30^ ARID1A suppression resulted in reduced non‐homologous end joining activity and sensitized cell lines to cytotoxic agents.^31^ However, the exact molecular mechanism of how loss of ARID1A expression causes paclitaxel resistant remains unclear. On the other hand, further studies are still needed to explore the role of *VMP1/MIR21* in the mechanism for the paclitaxel resistance of TNBCs as miR‐21 has been shown to be a useful biomarker to predict neoadjuvant therapeutic response in breast cancer patients.^32^

Evidence indicates that partial loss of ARID1A expression is significantly correlated with poor disease‐free survival in patients with invasive breast carcinoma. ARID1A down‐regulation significantly up‐regulated *RAB11* family interacting protein 1 (*RAB11FIP1*) mRNA in breast cancer cells.^33^ RAB11FIP1 is known as a Rab‐coupling protein that assists breast cancer progression.^34^ In the present study, *ARID1A* expression was up‐regulated in TNBCs compared to other subtypes derived from patients with breast cancer (Figure 3A‐D). *ARID1A* down‐regulation refers to a poor RFS rate in unclassified BRCA and TNBC patients (Figures 4 and S3). Furthermore, the mRNA levels of *ARID1A* in the high‐risk cohort are significantly down‐regulated compared to the low‐risk cohort in unclassified BRCA patients (Figure 4A). We also performed in silico analysis using IPA software to predict potentially activated/inhibited upstream regulators in TNBC cells. We found that p38MAPK and hydrogen peroxide were up‐regulated in DU4475 cells but were down‐regulated in MDA‐MB436 cells (Figure 6A and Table S2). The p38MAPK signalling pathway induces cell activation, proliferation and apoptosis.^35^ Recent studies have shown that the p38MAPK pathway is closely associated with drug resistance in cancer therapy. Sanchez‐Prieto et al indicated that inhibition of p38MAPK decreased the apoptotic fraction of cells exposed to chemotherapeutic agents and increased cell survival.^36^ Another recent study concluded that p38MAPK inhibition blocked p53‐dependent apoptosis allowing an autophagic response that mediated resistance.^37^ Previous studies have demonstrated that the antioxidant capacity of tumour cells scavenges excessive reactive oxygen species (ROS), allowing the disease to progress and develop resistance to apoptosis.^38^ Furthermore, toxic levels of ROS produced in cancers are anti‐tumorigenic, resulting in an increase in oxidative stress and induction of tumour cell death.^39^, ^40^ Here, we showed that *ARID1A* expression was positively correlated with the protein levels of pp38MAPK and highly regulated by p38MAPK‐related pathways in TNBCs.

In conclusion, the results of the present study demonstrated that *ARID1A* was predominantly up‐regulated upon paclitaxel treatment in TNBC cells that are sensitive to paclitaxel treatment. Furthermore, *ARID1A* expression was down‐regulated in breast cancer tissue compared to normal tissues. *ARID1A* down‐regulation predicts a significantly shorter RFS and poorer response to paclitaxel‐based chemotherapy in breast cancer. In addition, *ARID1A* expression was regulated by p38MAPK‐related signalling axis in the mechanism for paclitaxel resistance in TNBC. These observations further our understanding of the association between the *ARID1A* gene and drug resistance and may provide a novel therapeutic target for the treatment of TNBCs.

## CONFLICT OF INTEREST

The authors declare no conflict of interest.
