# Supplementary material for: High‐level expression of ARID1A predicts a favourable outcome in triple‐negative breast cancer patients receiving paclitaxel‐based chemotherapy
Source: J Cell Mol Med. 2018 Feb 1;22(4):2458–68. doi: 10.1111/jcmm.13551 (PMC5867090; doi:10.1111/jcmm.13551)
Supplement: Supplementary file 1 [file JCMM-22-2458-s001.docx]

**Supplementary Information**

**High-level expression of ARID1A predicts a favorable outcome in breast cancer patients receiving paclitaxel-based chemotherapy**

**Table of contents**

1. Table S1. List of consensus genes with 1.5-fold changes in MDA-MB436 and DU4475 cells following treatment with paclitaxel.
2. Table S2. List of consensus upstream regulators that are computationally predicted to be activated or inhibited after paclitaxel treatment in MDA-MB436 and DU4475 cells.
3. Figure S1. Transcriptional profiling of *ARID1A* and *VPM1/MIR21*in DU4475 and MDA-MB436 cells post-treatment without or with paclitaxel.
4. Figure S2. Correlations among different probes of *ARID1A* and *VMP1/MIR21* genes and PTX IC50 concentration in a panel of breast cancer cell lines without or with PTX treatment.
5. Figure S3. Prognostic significance of different *ARID1A* probes in K-M Plotter database against breast cancer patients.
6. Figure S4. Computational simulation of p38MAPK and hydrogen peroxide- targeting genes in paclitaxel-treated DU4475 and MDA-MB436 cells.

**Supplementary Table 1.** List of consensus genes with 1.5-fold changes in MDA-MB436 and DU4475 cells following treatment with paclitaxel.

| Probe ID | MB436 | DU4475 | Gene Symbol |
| --- | --- | --- | --- |
| 244766_at | 2.110755 | 1.050476055 | BOLA2 |
| 241387_at | 1.9241252 | 1.292438047 |  |
| 241242_at | 1.6772163 | 1.05111169 |  |
| 239121_at | 1.50925975 | 0.67206618 |  |
| 1559529_at | 1.432448893 | 1.030116 | PTK2 |
| 239937_at | 1.41765598 | 1.01064918 | ZNF207 |
| 224559_at | 1.38089655 | 1.20180802 | MALAT1 |
| 239228_at | 1.3473945 | 0.88147734 |  |
| 239892_at | 1.28810405 | 0.61554694 |  |
| 243046_at | 1.259673095 | 0.654328334 |  |
| 242751_at | 1.25759122 | 0.5825739 |  |
| 235959_at | 1.255675593 | 0.62685825 |  |
| 234989_at | 1.2553625 | 0.8262796 | MIR612 |
| 215190_at | 1.2378445 | 0.61431932 | EIF3M |
| 242261_at | 1.23605489 | 0.91750073 | IREB2 |
| 238156_at | 1.2323322 | 0.6249876 |  |
| 1552729_at | 1.20111088 | 0.65270326 | SNHG7 |
| 215123_at | 1.185637 | 0.64108372 | LOC101929910 |
| 242431_at | 1.17493439 | 0.66911506 |  |
| 229514_at | 1.16498684 | 0.64554214 | GPATCH2L |
| 243751_at | 1.16245344 | 0.763166 | CHD2 |
| 214782_at | 1.16132546 | 0.941082 | CTTN |
| 242265_at | 1.14488076 | 0.88452053 | BRD8 |
| 225239_at | 1.14083862 | 0.5874581 | MIR612 |
| 223494_at | 1.13935943 | 0.597760225 | MGEA5 |
| 214805_at | 1.136024 | 0.763542178 | EIF4A1 |
| 1569181_x_at | 1.101217 | 0.6607723 |  |
| 238951_at | 1.08766216 | 0.58804008 |  |
| 244659_at | 1.07324888 | 0.704446328 | TRIP12 |
| 244341_at | 1.07258467 | 0.80173873 |  |
| 1558732_at | 1.05747941 | 1.2146206 | MAP4K4 |
| 244427_at | 1.05058096 | 0.87950039 | KIF23 |
| 207331_at | 1.03951215 | 1.54050507 | CENPF |
| 1559993_at | 1.035898685 | 0.65235663 | SFXN3 |
| 1566901_at | 1.0329771 | 0.97404142 | TGIF1 |
| 240452_at | 1.01990082 | 0.76877593 | GSPT1 |
| 243037_at | 1.0187955 | 0.77641536 |  |
| 235646_at | 1.00559854 | 0.802872185 |  |
| 232466_at | 1.00551752 | 0.95458267 | /CUL4A |
| 243514_at | 0.9852319 | 0.7251625 | WDFY2 |
| 239243_at | 0.980352425 | 0.83107235 | ZNF638 |
| 230761_at | 0.96719933 | 0.61989643 |  |
| 1559490_at | 0.966027029 | 0.75403881 | LRCH3 |
| 226840_at | 0.959691527 | 0.63331704 | H2AFY |
| 209446_s_at | 0.9544678 | 0.61626386 |  |
| 1558783_at | 0.9498372 | 0.740065575 |  |
| 235716_at | 0.94620083 | 0.638913628 |  |
| 1557804_at | 0.94306825 | 0.86028766 |  |
| 1556277_a_at | 0.93566179 | 0.83877089 | PAPD4 |
| 236431_at | 0.92137477 | 0.81085157 | U2SURP |
| 236907_at | 0.90249156 | 1.09789893 |  |
| 242362_at | 0.8792906 | 0.596317287 |  |
| 238988_at | 0.87890577 | 0.83261346 |  |
| 215013_s_at | 0.86175345 | 0.61565065 | USP34 |
| 233595_at | 0.85507872 | 0.93325996 | USP34 |
| 244846_at | 0.85062123 | 0.70700116 |  |
| 235493_at | 0.84917258 | 0.65621332 |  |
| 228729_at | 0.84475708 | 0.59934044 | CCNB1 |
| 240451_at | 0.84380508 | 0.74833725 |  |
| 215645_at | 0.83806181 | 0.614024662 | FLCN |
| 233248_at | 0.8344598 | 0.89231107 |  |
| 233713_at | 0.754127495 | 0.6643253 |  |
| 1558750_a_at | 0.7523398 | 0.6046467 | ARHGAP11B |
| 240247_at | 0.73781872 | 0.74595593 |  |
| 1556007_s_at | 0.73248578 | 0.81449414 | CSNK1A1 |
| 242688_at | 0.72706796 | 0.720472822 |  |
| 215636_at | 0.71677542 | 0.60238409 | UBR4 |
| 244778_x_at | 0.70813369 | 0.82090093 |  |
| 215942_s_at | 0.70082523 | 0.834526066 | GTSE1 |
| 1565703_at | 0.68181276 | 0.63661717 | SMAD4 |
| 235456_at | 0.6678181 | 1.092747675 |  |
| 238797_at | 0.6556091 | 0.72614052 | TRIM11 |
| 235425_at | 0.64351654 | 0.62944033 | SGOL2 |
| 209283_at | 0.6396523 | 0.7459908 | CRYAB |
| 236193_at | 0.6268279 | 0.71641755 | HIST1H2BC |
| 244869_at | 0.6258111 | 0.8759496 |  |
| 237600_at | 0.61327028 | 0.58058738 |  |
| 232865_at | 0.599380975 | 0.6292243 | AFF4 |
| 242726_at | 0.59898187 | 0.87960527 |  |
| 239930_at | 0.59642316 | 0.61269327 | GALNT2 |
| 212142_at | -0.6041155 | -0.87101363 | MCM4 |
| 1553120_at | -0.6066465 | -0.6413183 | CLSPN |
| 210649_s_at | -0.615580561 | 0.646596 | ARID1A |
| 233230_s_at | -0.636070745 | -0.6159792 | SLAIN2 |
| 215220_s_at | -0.63694333 | -0.78768156 | TPR |
| 242878_at | -0.6513181 | -0.6592321 |  |
| 211814_s_at | -0.6827083 | -0.8429127 | CCNE2 |
| 33148_at | -0.6847215 | -0.954576 | ZFR |
| 224917_at | -0.697604176 | 0.6197872 | MIR21 |
| 1553122_s_at | -0.70760917 | -0.81059501 | RBAK |
| 202118_s_at | -0.7486334 | -0.6194482 | CPNE3 |
| 1557129_a_at | -0.8511028 | -0.7067132 | FAM111B |
| 211450_s_at | -0.93170165 | -0.6465712 | MSH6 |

**Supplementary Table 2.** List of consensus upstream regulators that are computationally predicted to be activated or inhibited after paclitaxel treatment in MDA-MB436 and DU4475 cells.

| MB436_Upstream Regulators | Activation z-score | p-value of overlap | DU4475_Upstream Regulators | Activation z-score | p-value of overlap |
| --- | --- | --- | --- | --- | --- |
| cephaloridine | -3.606 | 1.70E-03 | cephaloridine | -2.804 | 1.21E-01 |
| hydrogen peroxide | -2.724 | 1.32E-02 | hydrogen peroxide | 2.048 | 3.59E-08 |
| let-7a-5p | 2.34 | 1.94E-02 | let-7a-5p | 2.058 | 3.18E-01 |
| miR-16-5p | 2.226 | 7.62E-07 | miR-16-5p | 2.361 | 6.35E-02 |
| mir-181 | 2.408 | 1.59E-02 | mir-181 | 2.213 | 3.35E-02 |
| P38 MAPK | -2.285 | 3.93E-04 | P38 MAPK | 3.658 | 9.83E-02 |
| PLK2 | -2.236 | 8.12E-03 | PLK2 | -2 | 2.86E-02 |
| PLK4 | -2.236 | 6.51E-03 | PLK4 | -2 | 2.42E-02 |
| PTGER2 | 2.714 | 3.98E-05 | PTGER2 | 2.72 | 1.11E-02 |
| STAT3 | 2.375 | 9.91E-08 | STAT3 | 2.099 | 1.23E-02 |


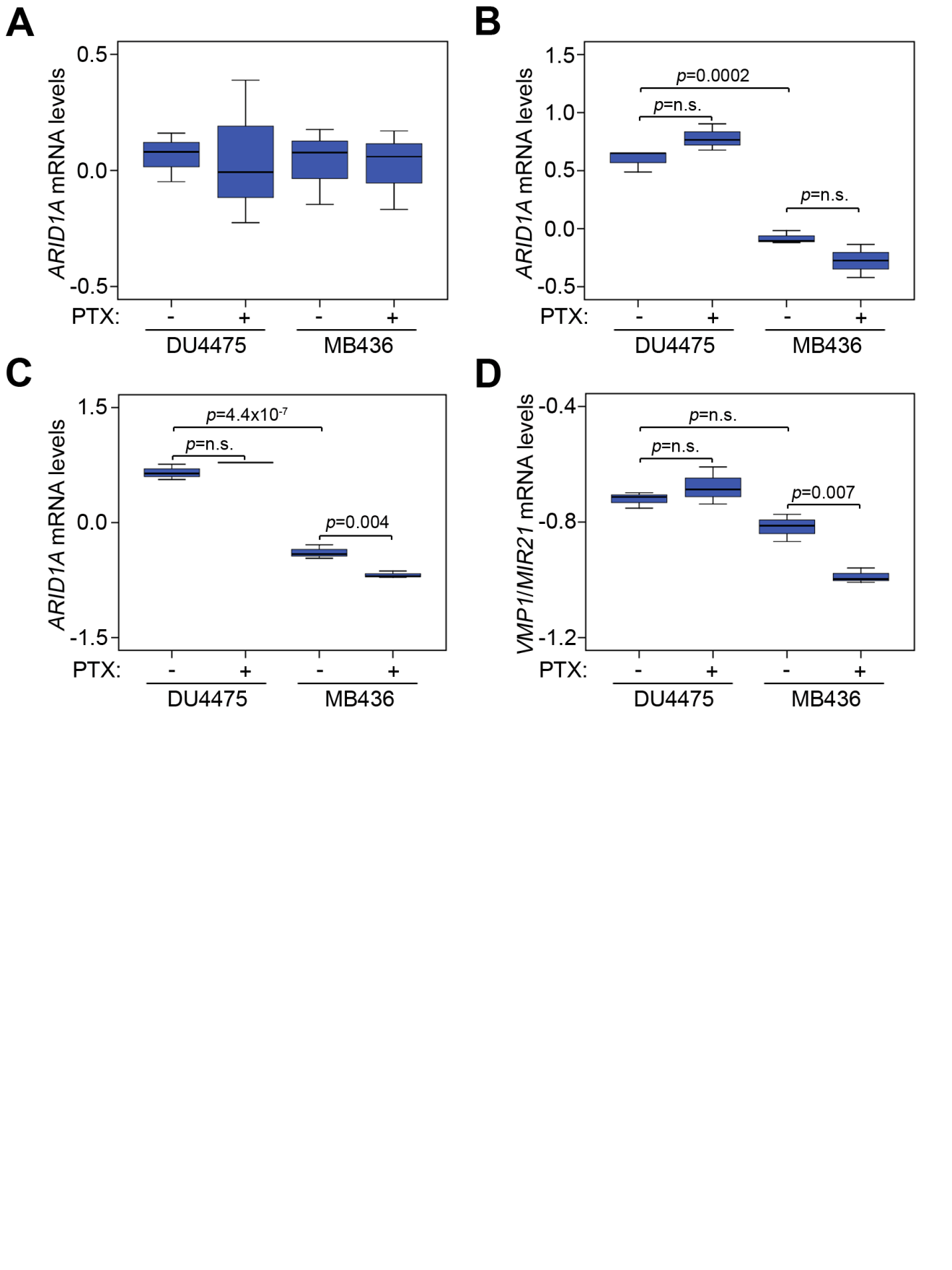


**Supplementary Figure 1.** Transcriptional profiling of *ARID1A* and *VPM1/MIR21*in DU4475 and MDA-MB436 cells post-treatment without or with paclitaxel. (A-C) *ARID1A* mRNA levels detected by different probes 207591_s_at (A), 212152_x_at (B) and 218917_s_at (C) in microarray dataset (GSE58032) against DU4475 and MDA-MB436 cells post-treatment without or with paclitaxel at the concentration of 10 x IC_50_ for 24 hours. (D) *VPM1/MIR21* mRNA levels detected by probe 220990_s_at in the same condition as above. Data from three independent experiments were shown in median ± SD. The statistical differences were analyzed by One-way ANOVA using Turkey’s test.


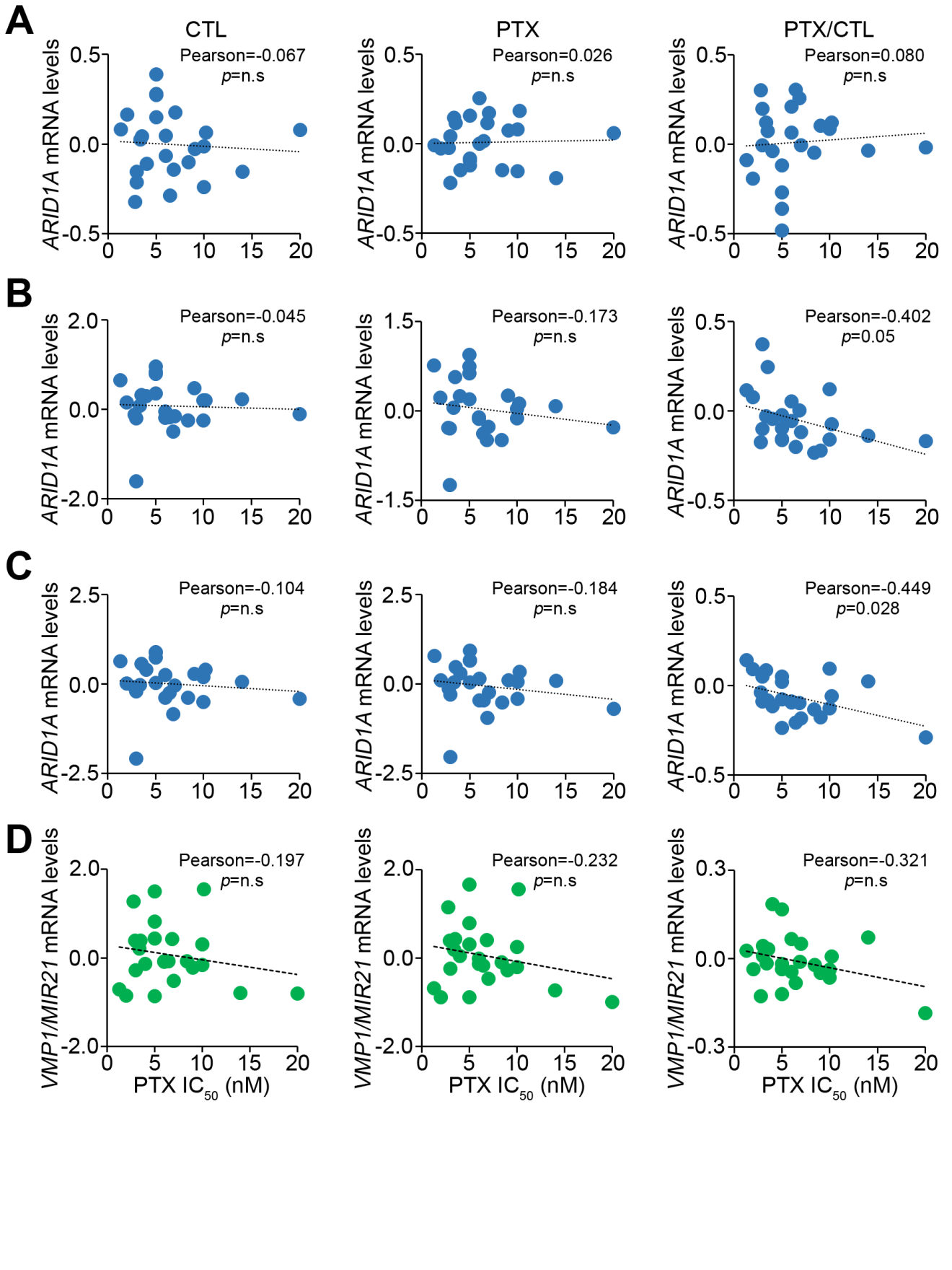


**Supplementary Figure 2.** Correlations among different probes of *ARID1A* and *VMP1/MIR21* genes and PTX IC50 concentration in a panel of breast cancer cell lines without or with PTX treatment. (A–C) Correlations among *ARID1A* probes 207591_s_at (A), 212152_x_at (B) and 218917_s_at (C) in GSE50832 dataset and PTX IC50 in tested breast cancer cell lines without (control, CTL) or with PTX treatment. (D) Correlation of *VMP1/MIR21* probe 220990_s_at in GSE50832 dataset with PTX IC50 in tested breast cancer cell lines without or with PTX treatment.


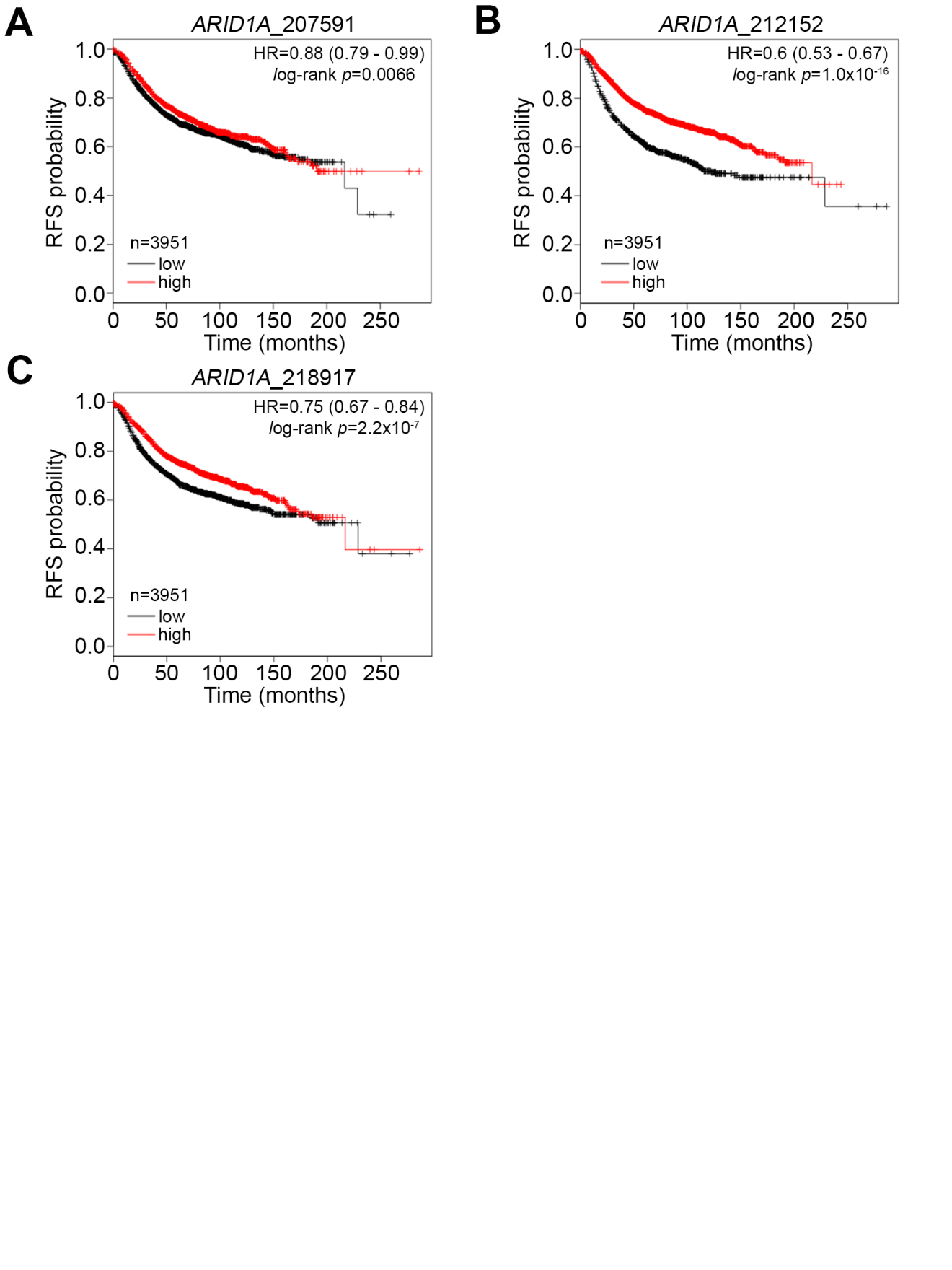


**Supplementary Figure 3.** Prognostic significance of different *ARID1A* probes in K-M Plotter database against breast cancer patients. (A–C) Prognostic estimation of *ARID1A* probes 207591_s_at (A), 212152_x_at (B) and 218917_s_at (C) towards survival rates of breast cancer patients under the condition of recurrence-free survival (RFS) probability by using K-M Plotter database. HR denotes hazard ration at 95% confidence interval.


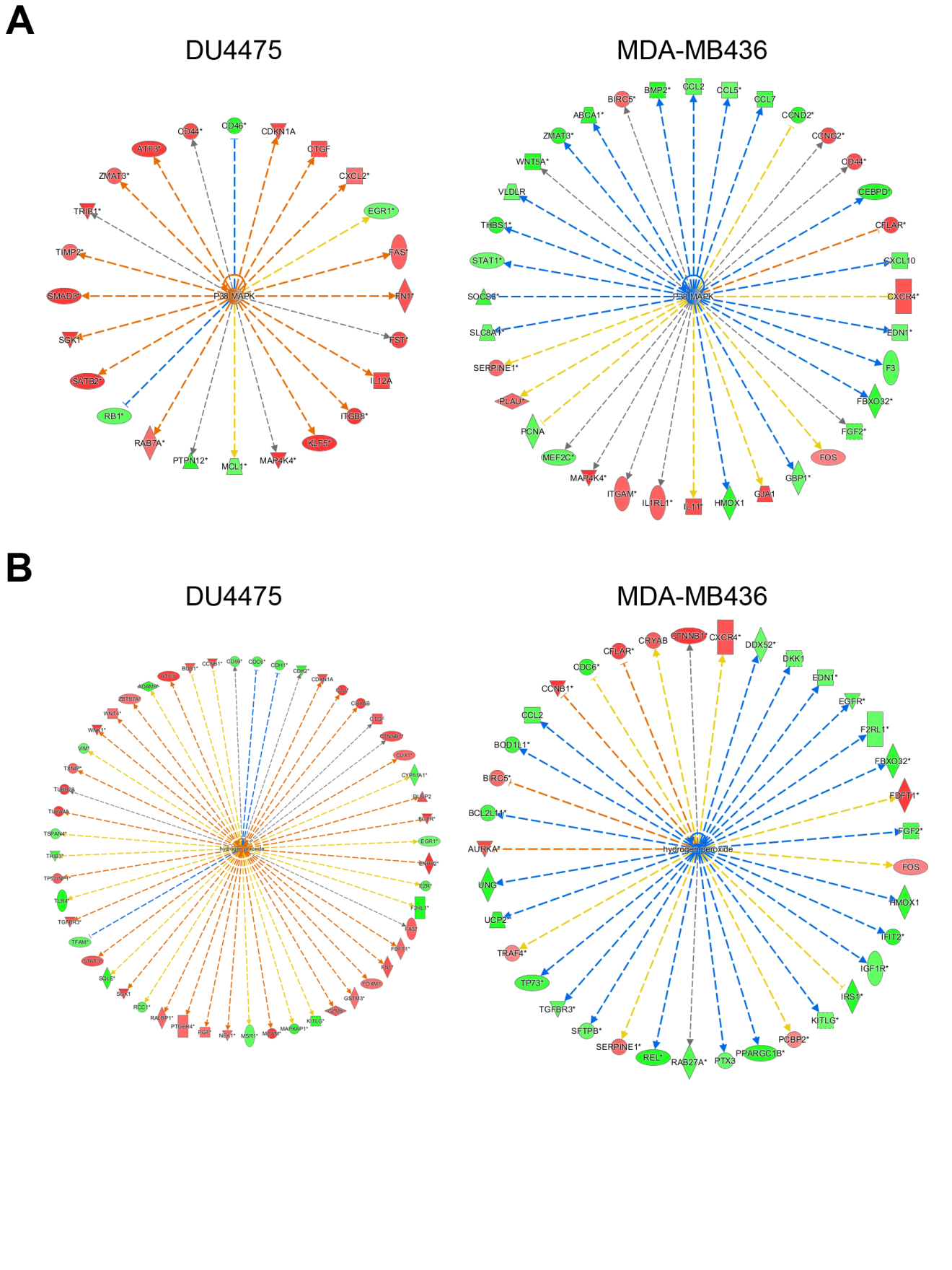


**Supplementary Figure 4.** Computational simulation of p38MAPK and hydrogen peroxide- targeting genes in paclitaxel-treated DU4475 and MDA-MB436 cells. (A and B) Computational simulation of p38MAPK (A) and hydrogen peroxide (B)-targeting genes in DU4475 and MDA-MB436 cells following treatment with paclitaxel at 10 x IC_50_ for 24 hours.
